# Contributing factors for intraocular pressure control in patients with mostly normal-tension glaucoma after initial Ex-PRESS drainage device implantation

**DOI:** 10.1007/s00417-023-06209-8

**Published:** 2023-08-25

**Authors:** Yurika Aoyama, Rei Sakata, Takashi Fujishiro, Megumi Honjo, Shiroaki Shirato, Makoto Aihara

**Affiliations:** 1 Department of Ophthalmology, Graduate School of Medicine and Faculty of Medicine, The University of Tokyo, Tokyo, 113-8655 Japan; 2 Yotsuya Shirato Eye Clinic, Tokyo, Japan

**Keywords:** Ex-PRESS, Intraocular pressure, Primary open-angle glaucoma, Glaucoma surgery, Bleb needling

## Abstract

**Purpose:**

To investigate the postoperative intraocular pressure (IOP) control and identify the factors associated with failure of initial Ex-PRESS surgery in patients with open-angle glaucoma for 3 years.

**Methods:**

A total of 79 patients with medically uncontrolled open-angle glaucoma (55 normal-tension glaucoma and 24 primary open-angle glaucoma) were enrolled. All patients underwent Ex-PRESS implantation (including combined cataract surgery). The outcome measure was the survival rate using life table analysis, the failure was defined as IOP of > 18 mmHg (criterion A), > 15 mmHg (criterion B) or > 12 mmHg (criterion C) and/or IOP reduction of < 20% from baseline (each criterion) without any glaucoma medications. The Cox proportional hazards model was used to identify risk factors for IOP management defined as the above criterion.

**Results:**

The mean preoperative IOP was 19.3 ± 5.8 mmHg. At 36 months, the mean IOP was 11.8 ± 3.6 mmHg with a mean IOP change of 7.5 mmHg (reduction rate 39.0%). The cumulative probability of success was 58% (95%CI: 42–64%) (criterion A), 48% (95%CI: 37–59%) (criterion B) and 30% (95%CI: 20–40%) (criterion C). In multivariate analyses, factors that predicted poor IOP control included the intervention of bleb needling after 6 months after the surgery (HR: 2.43; 95%CI: 1.35–4.37; *P* = 0.032). Transient hypotony was observed in 4 patients.

**Conclusion:**

The implementation of bleb needling after Ex-PRESS surgery in the late postoperative period was suggested to be the main risk factor for achieving lower IOP.


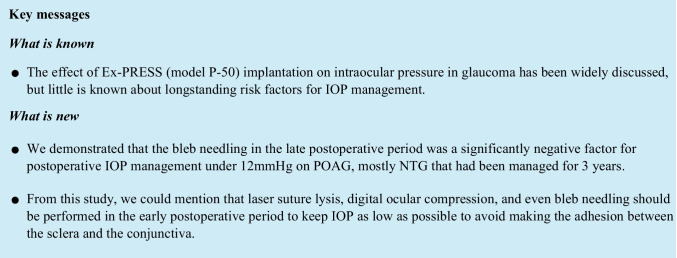


## Introduction

Trabeculectomy with mitomycin C (MMC) remains the core procedure in glaucoma filtration surgery [1, 2]. However, Ex-PRESS (Alcon, Fort Worth, TX, USA), a stainless-steel glaucoma drainage device with a total length of 2.64 mm and a lumen of 50 µm without a pressure regulating valve (called model P-50, available in Japan from 2011), is another filtration surgical procedure [3]. After a scleral flap is created in a procedure similar to trabeculectomy, Ex-PRESS is inserted into the anterior chamber to filter the aqueous humour into the subconjunctiva instead of performing a scleral block resection [4]. Ex-PRESS insertion technique does not require peripheral iridectomy, which results in shorter intraoperative anterior chamber opening time, less frequent postoperative anterior chamber haemorrhage, less postoperative inflammation and faster visual recovery [5]. Additionally, because the aqueous humour is filtered through the Ex-PRESS lumen, the amount of aqueous humour filtration is reported to be almost constant and there is less variation in postoperative IOP and a lower rate of postoperative hypotony than those of trabeculectomy. Thus, Ex-PRESS was selected not only for normal glaucoma but also for refractory patients with no vitreous or extremely high myopia. A recent meta-analysis based on four randomised controlled trials of 292 eyes confirmed that Ex-PRESS implantation and trabeculectomy have similar efficacy in intraocular pressure (IOP) reduction, medication reduction, vision recovery and qualified operative success rates [6]. Ex-PRESS implantation was associated with higher rates of complete operative success and fewer hyphemia cases than trabeculectomy.

Long-term results of Ex-PRESS (model P-50) have been reported and tracked over a period of 6–28 months, both domestically [7–11] and internationally [5, 12–15]. Although the baseline IOP varies in these studies, the mean postoperative IOP reduction rate is around 44.4%, the success rate of IOP management is approximately 80% when the IOP of < 18 mmHg and the rate of IOP reduction of 20% is adopted. The failure of Ex-PRESS shunts implanted under a scleral flap is normally a consequence of episcleral or bleb scarring [16], despite the use of antimetabolites. The growth of fibrotic tissue between the device and the scleral flap has been grown in vivo [17], indicating one of the failure mechanisms. One study evaluating the bleb morphology, not IOP, after Ex-PRESS (model P-50) in Japanese patients with glaucoma, found that advanced age, higher postoperative IOP, pseudoexfoliation and simultaneous cataract surgery negatively affected bleb functioning [18].

Although 20 years have passed since Ex-PRESS became available, few reports are available on the results of surgery using model P-50 Ex-PRESS only in a large number of primary open-angle glaucoma (POAG) eyes and risk factors for IOP management have not been examined. Therefore, this study aimed to investigate the postoperative IOP course of initial Ex-PRESS (model P-50) for POAG including normal-tension glaucoma (NTG, more than half) and risk factors that affect IOP control.

## Subjects and methods

The medical records of Japanese patients with glaucoma who underwent an initial Ex-PRESS at Yotsuya Shirato Eye Clinic (Tokyo, Japan) between March 2014 and June 2016 were retrospectively evaluated. All protocols and methods followed the tenets of the Declaration of Helsinki. This study was approved by the Ethics Committee of Riverside Internal Medicine Cardiology Clinic (approval ID: RSC-1811RB01). Informed consent was waived by the Research Ethics Board due to the retrospective nature of the study. The inclusion criteria were as follows: patients with POAG, patients who underwent Ex-PRESS alone or combined cataract surgery and could be followed up for 36 months and eye with no contact of the Ex-PRESS tip with the corneal endothelium confirmed by gonioscope during the course. The exclusion criteria were as follows: patients with primary angle-closure glaucoma, patients with secondary glaucoma caused by uveitis, pseudoexfoliation or steroid, patients with a history of laser iridotomy, patients who underwent an additional surgery involving the conjunctiva or vitreous and eyes requiring another glaucoma surgery after Ex-PRESS.

### Ophthalmic examinations

Slit lamp and fundus examinations were routinely conducted at each visit. IOP was measured using a calibrated Goldmann applanation tonometer (Haag-Streit, Wedel, Germany) during office hours after administering topical anaesthesia in a sitting position. Refractive error was measured using an autorefractor keratometer (ARK-900, Nidek, Gamagori, Japan). The relationship between the tip and the corneal endothelium or iris was checked using a gonioscope with a Sussman 4-mirror lens every six months.

### Visual field (VF) test

VF tests were performed at least four times during the course (at least once a year), using a Humphrey Visual Field Analyser (HFA) (Carl Zeiss Meditec, Jena, Germany) according to the standard 30–2 programmes of the Swedish Interactive Threshold Algorithm (SITA) with reliability indices of the VF test as follows: fixation, < 20%; false-positive rate, < 15%; and false-negative rate, < 15%.

### Surgical technique

Ex-PRESS surgery (including combined cataract surgery) was performed by two well-trained surgeons (SS and MA). The surgical technique was as follows. After instilling anaesthesia into the sub-Tenon space, a 5- to 6-mm fornix-based conjunctival incision was made along the limbus at the temporal or nasal side. This process was followed by cauterisation, formation of a single rectangular scleral flap, soaking with 0.05% MMC for 1.5 min and washout of residual MMC with 100 mL of a balanced salt solution. A 25-gauge needle was inserted from the scleral floor to the anterior chamber at the anatomical ring (grey zone), followed by Ex-PRESS (model P-50) insertion, release and fixation at the same site. Then, placement of two or three 10–0 nylon scleral flap sutures, adjustment of aqueous humour flow with an occasional additional suture and placement of a wing conjunctival suture with ophthalmic 10–0 nylon sutures (MANI, Utsunomiya, Japan) were performed. In combined cataract surgery cases, a clear corneal incision was made at the superior quadrant with viscoelastic materials (Viscoat 0.5 Ophthalmic Viscoelastic Substance [Alcon, Tokyo, Japan] and 1% Healon Ophthalmic Viscoelastic Substance [AMO, Tokyo, Japan]) using the soft-shell technique. Postoperative medical treatment consisted of four applications of 0.1% betamethasone and moxifloxacin ophthalmic solution. For the combined surgery, two applications of diclofenac sodium ophthalmic solution were added. If IOP was elevated after the surgery, either laser suture lysis (Blumenthal suture lysis lens, Volk) or bleb needling was performed at the surgeon’s discretion to increase the flow rate of the aqueous humour.

### Statistical analysis

The first operated eye was selected if both eyes met the inclusion criteria. The amount of IOP change after Ex-PRESS was evaluated in the case of the whole and for each surgical procedure (single or combined). The change in IOP during the course was evaluated by analysis of variance performed using a mixed model. The amount of IOP decrease was calculated as follows: Preoperative IOP–postoperative IOP and the IOP reduction rate (%) was calculated as follows: (preoperative IOP–postoperative IOP)/preoperative IOP) × 100. The survival analysis of IOP management at 36 months was examined by the Kaplan–Meier (KM) survival curve. The definition of failure in the KM curve was set in three cases, that is, IOP of > 18 mmHg and/or < 20% IOP reduction from preoperative IOP without any glaucoma medications (criterion A), IOP of > 15 mmHg and/or < 20% IOP reduction from preoperative IOP without any glaucoma medications (criterion B) and IOP of > 12 mmHg and/or < 20% IOP reduction from preoperative IOP without any glaucoma medications (criterion C). These cut-off values were based on previous reports [19]. Reoperation and removal of Ex-PRESS were also judged as a failure in all criteria. The differences in the surgical procedures were compared using the log-rank test. This time, failure was assumed if the above-mentioned cut-off value was reached even once rather than twice. Univariate and multivariate Cox proportional hazard models were used to detect the risk factors of IOP management. Covariates with *P* < 0.1 in univariate analysis proceeded in multivariate analysis. Covariates included age, gender, refraction, preoperative lens status (phakia or pseudophakia), glaucoma disease type (NTG or POAG), surgical technique (single or combined), preoperative IOP, preoperative medication score, the number of laser suture lysis procedures and the implementation of bleb needling (within 6 months of the surgery or later). Mean deviation (MD) and pattern standard deviation (PSD) values at the preoperative and postoperative at 36 months were compared using a *t*-test. Complications, such as hypotony (IOP < 5 mmHg), bleb leak, hyphema, choroidal detachment, infection and device-related events (visible obstruction), were assessed during the follow-up period. All statistical analyses were performed using JMP Pro (version 16.0, SAS Institute, Cary, NC, USA) and IBM SPSS Statistics (version 23.0, IBM Corp., Armonk, NY, USA) software. *P* < 0.05 was considered statistically significant unless otherwise specified.

## Results

A total of 79 patients with 79 eyes were included in the study. Among them, 46 were males and 33 were females, with a mean age of 63.3 ± 11.0 years. The patients’ background is shown in Table 1. Of the 79 eyes, 24 underwent Ex-PRESS surgery alone and 55 underwent simultaneous phacoemulsification and intraocular lens (IOL) insertion.

**Table 1:** Clinical characteristics of patients

	Total (79 eyes)	Single (24 eyes)	Combined (55 eyes)
Age (years)	63.3 ± 11.0	60.9 ± 15.0	64.4 ± 8.6
Gender (M/F)	46/33	12/12	34/21
Diagnosis (number)			
POAG	36	12	24
NTG	43	12	31
Lens status (number)			
Phakia	63	8	55
Pseudophakia	16	16	0
Refraction (diopters)*	− 6.7 ± 4.8	− 7.9 ± 2.1	− 6.5 ± 5.0
Baseline IOP (mmHg)	19.3 ± 5.9	18.9 ± 7.4	19.5 ± 5.1

*M*, male; *F*, female; *POAG*, primary open-angle glaucoma; *NTG*, normal-tension glaucoma; *IOP*, intraocular pressure

^*^Only in phakic eyes

**Fig. 1:**
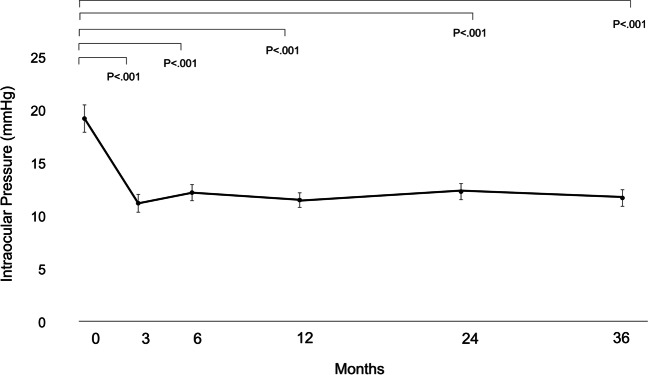
Changes in intraocular pressure after Ex-PRESS surgery. The horizontal axis represents the time course (months), and the vertical axis represents intraocular pressure (IOP: mmHg). The error bar represents the 95% confidential interval. A significant decrease was found in IOP at 3 months, 6 months, 12 months, 24 months and 36 months compared with the baseline

**Fig. 2:**
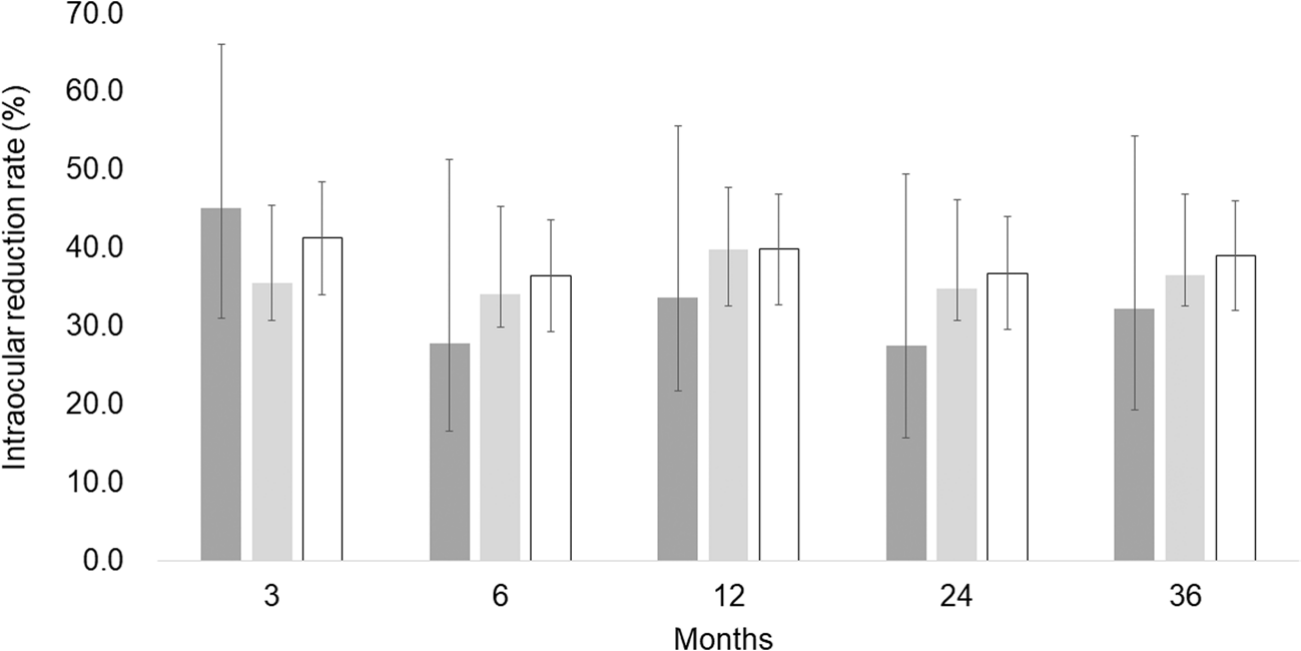
Changes in the reduction rate of intraocular pressure after Ex-PRESS surgery. The horizontal axis represents the time course (months), and the vertical axis represents the intraocular pressure (IOP) reduction rate (%) from preoperative IOP. The error bar represents the 95% confidence interval. Dark grey bars indicate single surgery, light grey bars indicate combined surgery and white bars indicate totality, respectively

**Fig. 3:**
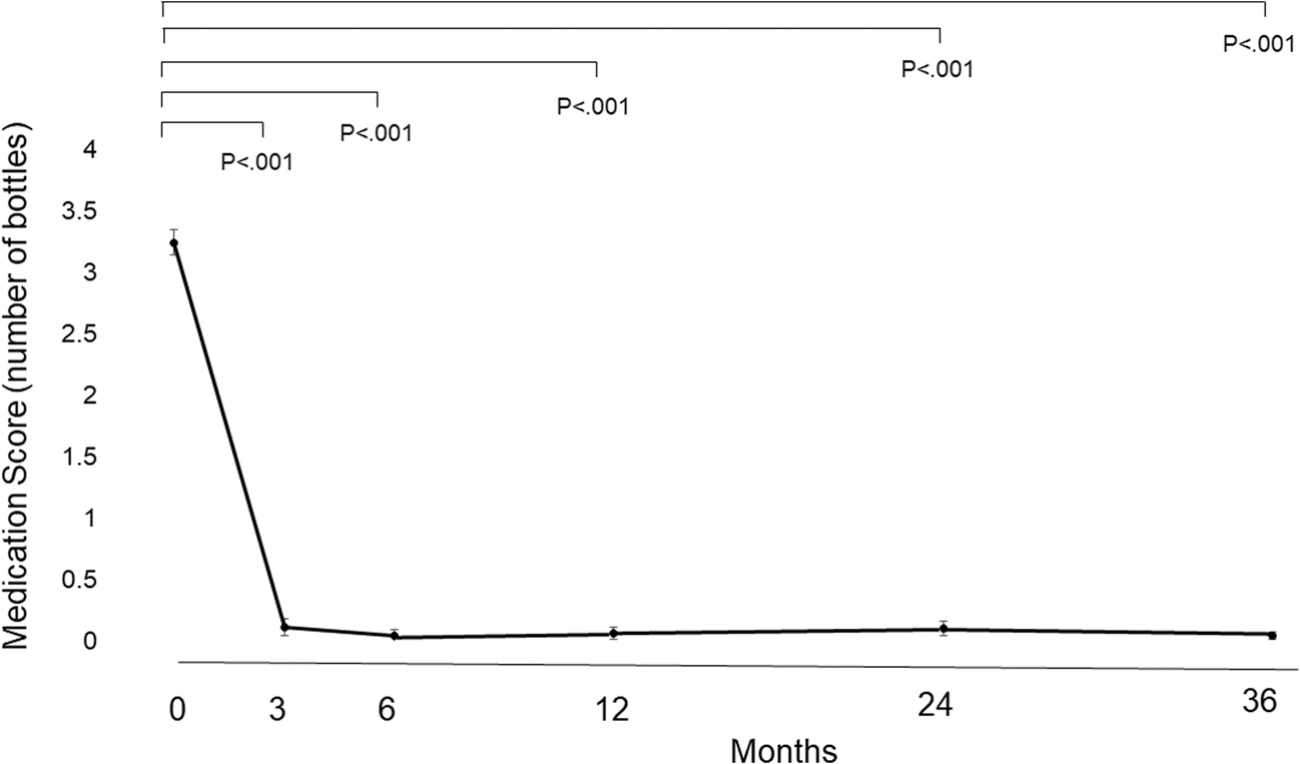
Changes in medication score after Ex-PRESS surgery. The horizontal axis represents the time course (months), and the vertical axis represents the number of medication score (number of bottles). The error bar represents the 95% confidential interval. A significant decrease was found in IOP at 3 months, 6 months, 12 months, 24 months and 36 months compared with the baseline

**Fig. 4:**
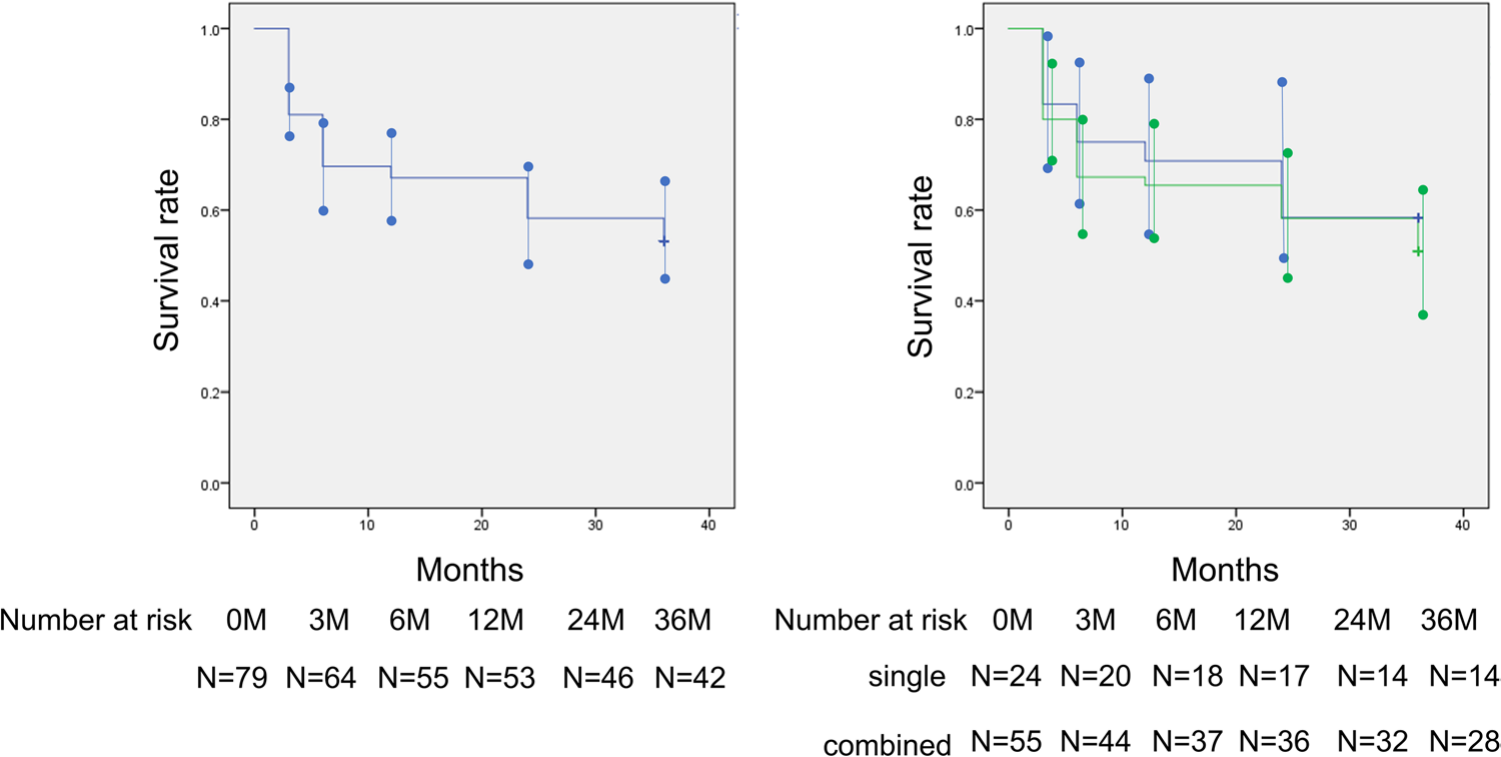
Kaplan–Meier survival analysis based on criterionA. Left: Kaplan–Meier survival analysis based on the definition of criterion A (definition of failure; IOP > 18 mmHg and/or < 20% IOP reduction) in all cases. The horizontal axis represents the time course (months), and the vertical axis represents the survival rate. The error bar represents the 95% confidential interval at each point. Right: Kaplan–Meier survival analysis based on the definition of criterion A of maintaining IOP divided by the surgical technique. The horizontal axis represents the time course (months), and the vertical axis represents the survival rate. The green line indicates combined cataract surgery, and the blue line indicates single surgery, with no difference between them (*P* = 0.57, log-rank test). The error bar represents the 95% confidential interval at each point

**Fig. 5:**
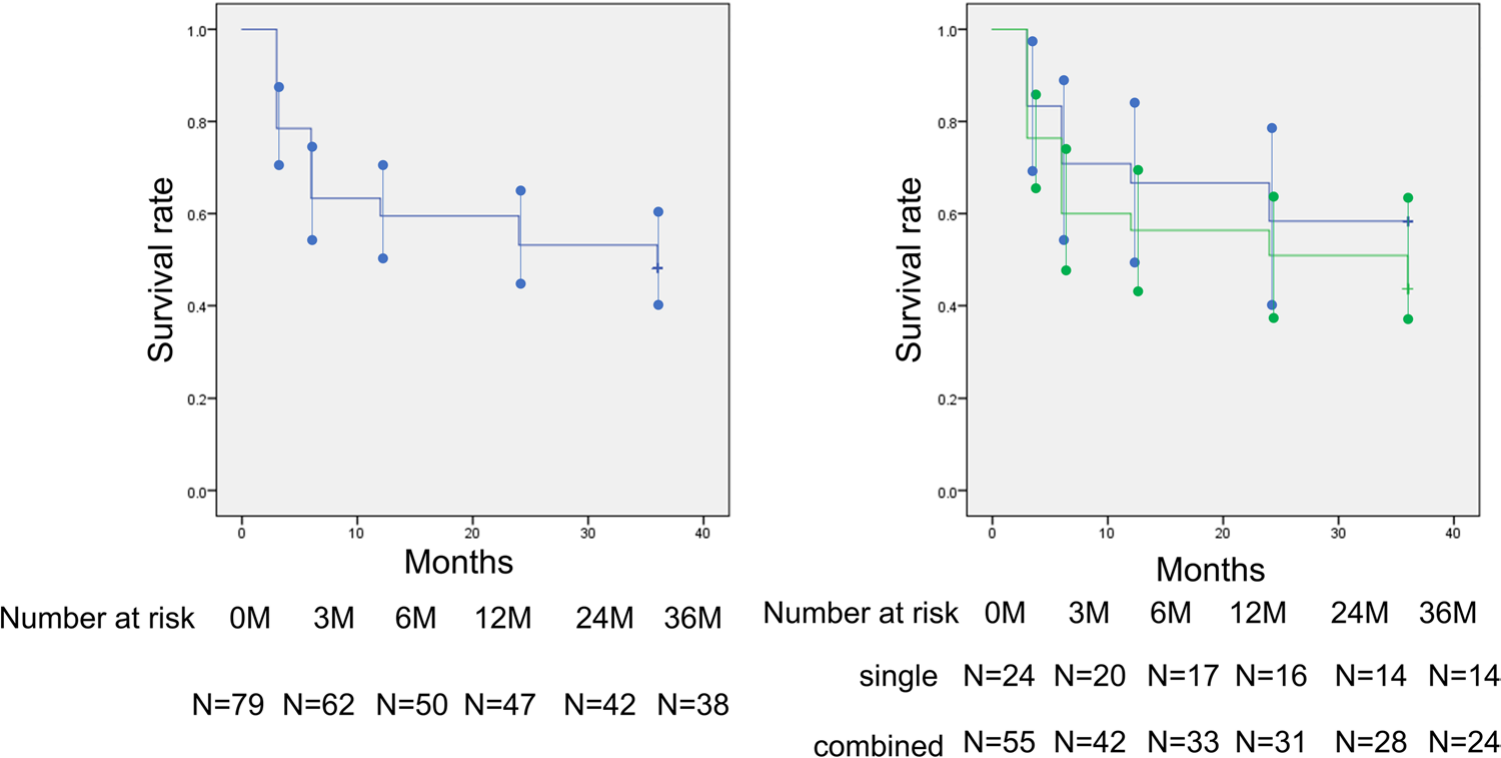
Kaplan–Meier survival analysis based on criterion B. Left: Kaplan–Meier survival analysis based on the definition of criterion B (definition of failure; IOP > 15 mmHg and/or < 20% IOP reduction) in all cases. The horizontal axis represents the time course (months), and the vertical axis represents the survival rate. The error bar represents the 95% confidential interval at each point. Right: Kaplan–Meier survival analysis based on the definition of criterion B of maintaining IOP divided by the surgical technique. The horizontal axis represents the time course (months), and the vertical axis represents the survival rate. The green line indicates combined cataract surgery, and the blue line indicates single surgery, with no difference between them (*P* = 0.26, log-rank test). The error bar represents the 95% confidential interval at each point

**Fig. 6:**
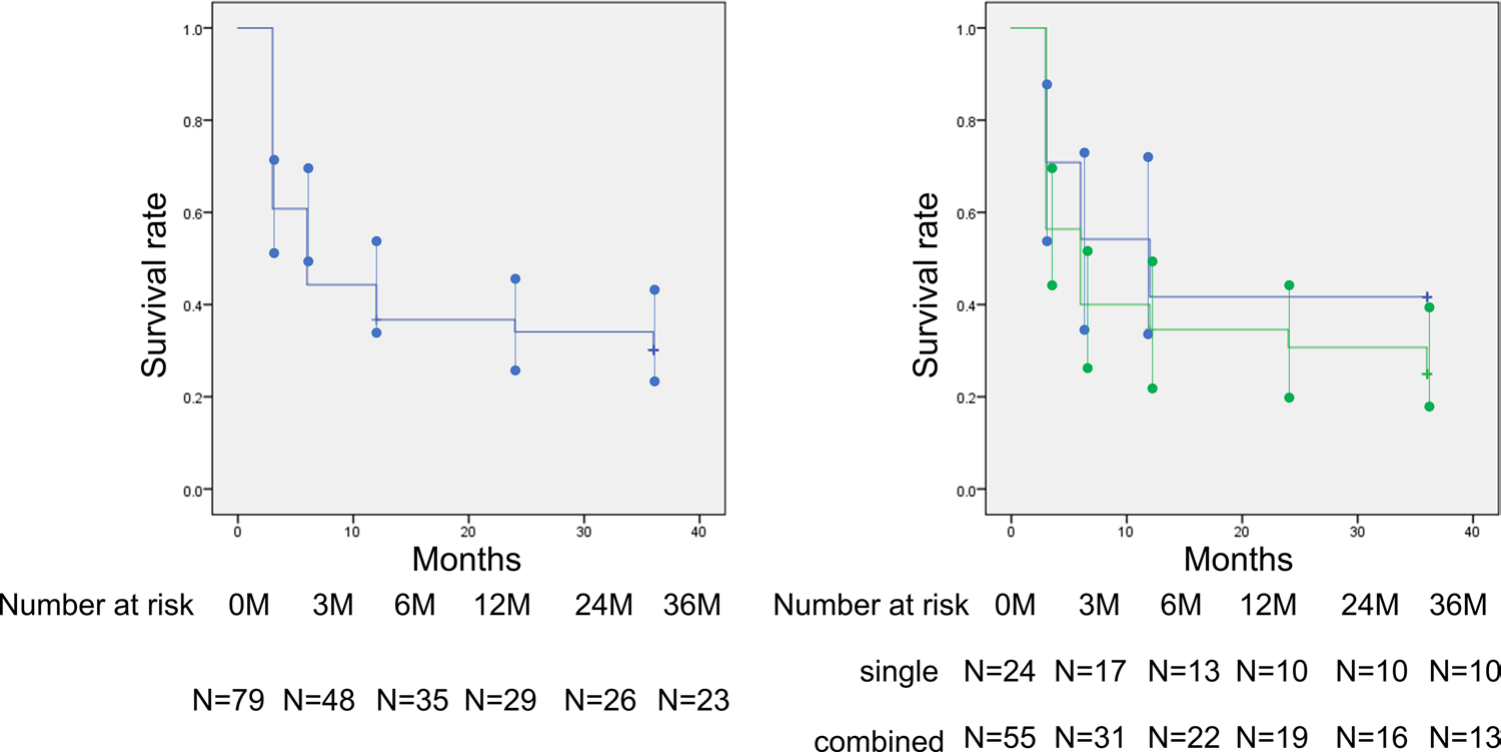
Kaplan–Meier survival analysis based on criterion C. Left: Kaplan–Meier survival analysis based on the definition of criterion C (definition of failure; IOP > 12 mmHg and/or < 20% IOP reduction) in all cases. The horizontal axis represents the time course (months), and the vertical axis represents the survival rate. The error bar represents the 95% confidential interval at each point. Right: Kaplan–Meier survival analysis based on the definition of criterion C of maintaining IOP divided by the surgical technique. The horizontal axis represents the time course (months), and the vertical axis represents the survival rate. The green line indicates combined cataract surgery, and the blue line indicates single surgery, with no difference between them (*P* = 0.16, log-rank test). The error bar represents the 95% confidential interval at each point

The mean preoperative IOP was 19.3 ± 5.8 mmHg. At 36 months postoperatively, the mean IOP was 11.8 ± 3.6 mmHg and the mean IOP change was 7.5 mmHg (mean reduction rate 39.0%) (Fig. 1). The mean number of laser suture lysis procedures was 1.82 ± 1.38 times, with no need for Nd:YAG laser treatment of the shunt tip. In the case of the single surgery, the IOP was 18.9 ± 7.3 mmHg preoperatively, and at 36 months postoperatively, the mean IOP was 12.0 ± 5.1 mmHg and the mean change was 7.0 mmHg (mean reduction rate 36.8%). Furthermore, in the combined cataract surgery, the mean IOP changed from 19.3 ± 5.1 mmHg to 11.7 ± 2.6 mmHg and the mean change was 7.8 mmHg (mean reduction rate 39.9%) (Table 2). The IOP reduction rate and mean medication score (overall) during the course are shown in Figs. 2 and 3. A total of 58 (15 for single surgery and 43 for combined surgery) and 44 (11 for single surgery and 33 for combined surgery) patients underwent one or more laser suture lysis and bleb needling procedures, respectively. In the survival analysis, the success rate at 36 months was 58% (95%CI: 42–64%) for criterion A (Fig. 4, left), with no difference by procedure (log-rank test, *P* = 0.57) (Fig. 4, right). Similarly, for criterion B, the success rate at 36 months was 48% (95%CI: 37–59%) (Fig. 5, left), with no difference by procedure (log-rank test, *P* = 0.26) (Fig. 5, right), and for criterion C, the success rate at 36 months was 30% (95%CI: 20–40%) (Fig. 6, left), with no difference by procedure (log-rank test, *P* = 0.16) (Fig. 6, right). The results of multivariate Cox proportional hazard analysis revealed that using criterion A (the least stringent criterion), it was relatively easier to control IOPs to < 18 mmHg in patients with POAG compared to the IOPs in those with NTG (Table 3). Criterion B did not reveal any significant factors (Table 4); however, criterion C (the strictest standard) suggested that IOP control would be poorer if bleb needling was performed after 6 months of the surgery (HR: 2.43, *P* = 0.032) (Table 5).

**Table 2:** Mean IOP before and after Ex-PRESS surgery

	IOP (mmHg)		
	Total (79 eyes)	Single (24 eyes)	Combined (55 eyes)
Baseline	19.3 ± 5.8 (95% CI:18.0 ~ 20.6)	18.9 ± 7.3 (95% CI:15.8 ~ 22.1)	19.5 ± 5.1 (95% CI:18.1 ~ 20.9)
3 months	11.3 ± 3.8 (95% CI:10.5 ~ 12.2)	9.7 ± 3.6 (95% CI:8.1 ~ 11.3)	12.0 ± 3.7 (95% CI:11.0 ~ 13.0)
6 months	12.3 ± 3.5 (95% CI:11.5 ~ 13.0)	12.5 ± 4.2 (95% CI:10.7 ~ 14.3)	12.2 ± 3.0 (95% CI:11.3 ~ 13.0)
12 months	11.6 ± 3.1 (95% CI:10.9 ~ 12.3)	11.6 ± 3.8 (95% CI:10.0 ~ 13.2)	11.6 ± 2.8 (95% CI:10.9 ~ 12.4)
24 months	12.2 ± 3.6 (95% CI:11.4 ~ 13.0)	12.8 ± 4.7 (95% CI:10.7 ~ 14.8)	12.0 ± 3.0 (95% CI:11.1 ~ 12.8)
36 months	11.8 ± 3.6 (95% CI:11.0 ~ 12.6)	12.0 ± 5.1 (95% CI:9.7 ~ 14.2)	11.7 ± 2.6 (95% CI:11.0 ~ 12.4)

*IOP*, intraocular pressure; *CI*, confidence interval

**Table 3:** Results of Cox proportional hazard model analysis of IOP management after Ex-PRESS surgery (criterion A)

Covariates	Univariate hazard ratio (95% CI)	*P* value	Multivariate hazard ratio (95% CI)	*P* value
Age	0.97 (0.95 ~ 1.00)	0.025	0.98 (0.96 ~ 1.01)	0.22
Gender (reference as male)	1.50 (0.76 ~ 2.95)	0.23		
Refraction	0.97 (0.92 ~ 1.04)	0.42		
Preoperative lens status (reference as pseudophakia)	3.39 (1.04 ~ 11.1)	0.016	2.69 (0.81 ~ 8.95)	0.11
Glaucoma type (reference as NTG)	0.35 (0.17 ~ 0.72)	0.004	0.39 (0.19 ~ 0.81)	0.012
Surgical technique (reference as single surgery)	1.21 (0.59 ~ 2.51)	0.59		
Preoperative IOP	0.93 (0.86 ~ 1.00)	0.041		
Preoperative medication score	1.40 (0.94 ~ 2.19)	0.098	1.20 (0.77 ~ 1.94)	0.44
Number of laser suture lysis	0.89 (0.70 ~ 1.13)	0.32		
Needling in the late postoperative period (reference as performed within 6 months after surgery)	1.71 (0.87 ~ 3.37)	0.12		

*NTG*, normal-tension glaucoma; *IOP*, intraocular pressure; *CI*, confidence interval

**Table 4:** Results of Cox proportional hazard model analysis of IOP management after Ex-PRESS surgery (criterion B)

Covariates	Univariate hazard ratio (95% CI)	*P* value	Multivariate hazard ratio (95% CI)	*P* value
Age	0.98 (0.95 ~ 1.00)	0.065	0.99 (0.96 ~ 1.02)	0.38
Gender (reference as male)	1.41 (0.75 ~ 2.67)	0.28		
Refraction	0.97 (0.91 ~ 1.03)	0.26		
Preoperative lens status (reference as pseudophakia)	3.87 (1.19 ~ 12.6)	0.006	3.13 (0.95 ~ 10.4)	0.061
Glaucoma type (reference as NTG)	0.54 (0.28 ~ 1.03)	0.062	0.60 (0.31 ~ 1.16)	0.13
Surgical technique (reference as single surgery)	1.45 (0.71 ~ 2.97)	0.29		
Preoperative IOP	0.99 (0.92 ~ 1.04)	0.65		
Preoperative medication score	1.41 (0.97 ~ 2.17)	0.070	1.24 (0.82 ~ 1.95)	0.33
Number of laser suture lysis	0.91 (0.72 ~ 1.14)	0.41		
Needling in the late postoperative period (reference as performed within 6 months after surgery)	1.41 (0.73 ~ 2.73)	0.31		

*NTG*, normal-tension glaucoma; *IOP*, intraocular pressure; *CI*, confidence interval

**Table 5:** Results of Cox proportional hazard model analysis of IOP management after Ex-PRESS surgery (criterion C)

Covariates	Univariate hazard ratio (95% CI)	*P* value	Multivariate hazard ratio (95% CI)	*P* value
Age	1.00 (0.98 ~ 1.02)	0.94		
Gender (reference as male)	1.22 (0.71 ~ 2.10)	0.47		
Refraction	0.98 (0.93 ~ 2.04)	0.52		
Preoperative lens status (reference as pseudophakia)	2.06 (0.93 ~ 4.57)	0.052	1.72 (0.77 ~ 3.88)	0.19
Glaucoma type (reference as NTG)	0.84 (0.49 ~ 1.43)	0.51		
Surgical technique (reference as single surgery)	1.44 (0.78 ~ 2.65)	0.23		
Preoperative IOP	1.03 (0.98 ~ 1.07)	0.33		
Preoperative medication score	1.17 (0.86 ~ 1.62)	0.33		
Number of laser suture lysis	1.00 (0.83 ~ 1.21)	1.00		
Needling in the late postoperative period (reference as performed within 6 months after surgery)	2.65 (1.48 ~ 4.73)	0.001	2.43 (1.35 ~ 4.37)	0.032

*NTG*, normal-tension glaucoma; *IOP*, intraocular pressure; *CI*, confidence interval

In 66 eyes (84%) in which a routine examination with the HFA30-2 was possible before and after surgery, the MD value was − 12.9 ± 10.9 dB before surgery and − 14.6 ± 11.0 dB at 3 years (*P* = 0.38), while the PSD value changed from 12.3 ± 2.8 dB to 11.9 ± 2.9 dB at 3 years (*P* = 0.04).

Postoperative complications included low postoperative IOP (*n* = 4), and transconjunctival scleral flap suture was performed in these patients. All had shallow anterior chambers but no choroidal detachment. The mean duration to suturing was 40.3 days from the operation, and all patients had increased IOP after the suture. Bleb leak (*n* = 1) was observed at 3 months. One patient developed central retinal vein occlusion at 34 months. No cases of anterior chamber haemorrhage, infection or device-related adverse events were reported.

## Discussion

This is the study with the longest follow-up period (36 months) and the largest number of patients (*n* = 79) reported using the model P-50 Ex-PRESS currently the most widely selected up to date [5, 7–9, 11–15]. We reported the 3-year progress of Ex-PRESS surgery in POAG including NTG eyes and investigated risk factors associated with IOP management. The results showed a significant reduction in both IOP and eye drop score after the surgery, with a mean IOP reduction rate of 39% at 3 years. The percentage of patients who maintained a postoperative IOP reduction of 12 mmHg or less and/or 20% or more from baseline IOP (without glaucoma eye drops) was 30%. Although transient postoperative over-filtration was observed in some cases, it was within the range that could be handled by transconjunctival scleral suturing at an outpatient clinic. These results indicate that it was possible to achieve favourable IOP control in most cases without major complications threatening visual acuity. The significant risk factor for failure to control IOP was the implementation of bleb needling after 6 months postoperatively.

The current standard for glaucoma filtration surgery is still trabeculectomy with MMC [20, 21]. This procedure is expected to delay glaucoma progression not only in high-tension glaucoma [22] but also in NTG [23–25]. However, postoperative complications are a major problem with this procedure, and the key to postoperative management is how to prevent these problems from occurring. In one study of 1240 cases, the complication rate was 47% (early complication rate), with the most frequent being hyphema (24.6%), shallow anterior chamber (23.9%), hypotony (24.3%) and bleb leak (17.6%) [6]. Although most resolved within a week or two after surgery, 18.8% lost visual acuity (> 1 Snellen line), mainly from cataract development. Additionally, 4.4% of patients suffered irreversible visual loss. Ex-PRESS surgery creates the same scleral flap as trabeculectomy, but it would provide more stable IOP control and safer postoperative management than the conventional method. Some studies have compared the surgical results of Ex-PRESS with those of trabeculectomy [12, 13, 26–29]. Among them, two randomised controlled trials have reported that the mean postoperative IOP, medication score and success rate are comparable at 1 to 2 years, with Ex-PRESS having faster postoperative visual recovery [12, 13]. Some other reports showed that postoperative IOP fluctuations and complications are less frequent with Ex-PRESS than with trabeculectomy. Although no long-term reports exist, the short-term efficacy and safety of the Ex-PRESS and trabeculectomy are considered almost equal and the Ex-PRESS is easy to use and offers advantages to the patient and surgeon both intraoperatively and postoperatively compared to conventional trabeculectomy.

There are two types of Ex-PRESS with lumen size, 200 μm (P-200) and 50 μm (P-50), but we will discuss previous reports [5, 7–9, 11–15] using the model P-50 that we used in this study. In a report on Japanese patients with glaucoma, in cases of POAG and secondary glaucoma, the Kaplan–Meier survival analysis revealed that the success rate was 100% at 1 year according to the definition of success (i.e. IOP between 5 and 21 mmHg with or without medications and no requirement of additional surgery or total loss of vision); furthermore, the IOP reduction rate was 46.5% [7]. For patients who exhibited different types of glaucoma (e.g. POAG, steroid-induced, neovascular, uveitic and chronic angle-closure glaucoma) with a preoperative IOP of 29.9 mmHg, the complete success rate after 2 years of surgery, defined by the postoperative IOP < 30% of the preoperative IOP, was 48.5%, and the IOP reduction rate was 44.1% [8]. In the case of POAG including NTG, the cumulative probabilities calculated by the Kaplan–Meier method were 77.8% ± 5.2% and 70.7% ± 6.2% at 12 months and 24 months, respectively, when the surgical success was defined as IOP of > 5 mmHg but < 15 mmHg and ≥ 20% reduction, without the need for additional glaucoma surgery, and the IOP reduction rate was 24.1% [9]. Finally, in the case of only NTG, the survival analysis defined clinical treatment failure as IOP of ≥ 12 mmHg or as < 20% reduction from baseline in two consecutive follow-up visits, and the cumulative survival rate at 12 months after surgery was 55.9% (IOP reduction rate: 31.1%) [11]. Each study defined survival differently, but in the present study, the strictest criterion was 30% and the most liberal criterion was 58%. This may be attributed to the longer follow-up period in our study than in previous studies. The IOP reduction rate in our study seemed to be similar to the previous report (IOP reduction rate: 39.0%). We must discuss not only the rate of IOP reduction but also the rate of IOP maintenance. The definition of IOP control rate varies from report to report, making simple comparisons difficult, but the report closest in the patients’ background to this study indicated 72% at 2 years (IOP of < 18 mmHg without glaucoma eye drops and > 20% reduction in IOP) [9]. Our study showed 58% at 3 years (same definition as above), but the decrease in the efficacy over time and the difference in the ratio of POAG to NTG in the patient population could be attributed to this difference. The postoperative IOP reduction rate of 40% or more was achieved in reports from overseas [5, 12–15], which we believe is due to the high preoperative IOP of 21 mmHg or higher. In this context, the success rate of surgery is generally reported to be around 80%. However, these reports included not only POAG but also exfoliation glaucoma, pigmentary glaucoma and chronic angle-closure glaucoma, with shorter follow-up periods of 0.5–2.3 years.

In Japan, there are many NTG with lower IOP and when the preoperative IOP was cut off at 15 mmHg and the high-teen (> 15 mmHg) and low-teen (≤ 15 mmHg) groups were compared, IOP management was better in the high-teen group than in the low-teen group at 3 years (data not shown). Thus, the indication for Ex-PRESS for eyes controlled at or below 15 mmHg under eye drops should be carefully determined based on age, stage of disease and other factors. Although the number of eyes with preoperative IOP of less than 15 mmHg was only 16 eyes, the mean IOP reduction at 3 years was 17%, with 4 eyes achieving an IOP equal to or greater than the preoperative IOP and 9 eyes achieving an IOP reduction rate of less than 20%. However, the mean medication score for these 16 eyes was 3.63 preoperatively and 0.07 postoperatively, suggesting that the IOP-lowering effect of Ex-PRESS for eyes with lower IOP was rather limited but may be considered in terms of eye drop management.

The risk factor analysis showed that the implementation of bleb needling at 6 months or later was suggested to be associated with poor IOP control. IOP management after Ex-PRESS is mainly handled by laser suture lysis, which can be performed as same as trabeculectomy. Since needling is a procedure to release scarring, not increasing the flow rate of Ex-PRESS, it might be more difficult to release adhesions by needling in the late postoperative period than in the early period, increasing the likelihood of poor IOP control. It seemed essential to prevent conjunctival adhesion by performing laser suture lysis and digital massage at an early stage within 6 months postoperatively before the adhesion becomes solid. Although the long-term follow-up after needling was not confirmed in this study, post-Ex-PRESS bleb reconstruction using a needle or knife is reported to be a safe and effective technique, and its results have been reported to be almost equivalent to those after trabeculectomy [30].

This study has some limitations. First, this was a retrospective study conducted at a single institution, and there may be a selection bias. Moreover, as we evaluated a limited number of participants, caution should be exercised while interpreting the results. Furthermore, we did not consider patients who underwent additional surgery because of complications or inadequate IOP reduction. In addition, generally, it is difficult to obtain stable data after surgery as many patients exhibit instability and transfer or drop out of the hospital. We also excluded cases that dropped out due to ocular complications for another intervention. Therefore, it is not possible to fully comprehend the usefulness of Ex-PRESS surgery from this study. Additionally, as most of the patients exhibited open-angle glaucoma with normal IOP, it would be difficult to apply the results of the present study outside of Asia, including Japan. The mean IOP reduction rate was 39% at 3 years, and if it was limited to eyes with NTG, the mean IOP reduction rate was 31% (40% for POAG). According to the collaborative normal-tension glaucoma study (CNTGS), a 30% IOP reduction rate is sufficient to delay the disease progression; thus, Ex-PRESS was adequately effective for NTG if the patient experienced no major postoperative problems.

Second, it is necessary to consider that the accuracy of Ex-PRESS surgery and postoperative results, including IOP management, might correlate to some extent with the years of surgical experience [31]. Therefore, the results of this study may not be applicable to all surgeons working with Ex-PRESS. Third, the evaluation of risk factors should include not only local ocular factors but also systemic factors. For example, although diabetes mellitus is mentioned as one of the significant risk factors [32], it is not practical to examine haemoglobin A1c (HbA1c) in all patients. Additionally, there may be cases where Ex-PRESS is selected after the failure of minimally invasive glaucoma surgery, which has been widely practiced recently. In such cases, as with cataract surgery, there should be a breakdown of the blood-aqueous barrier [33], so the effectiveness of Ex-PRESS needs to be further studied and more cases need to be accumulated. Finally, the goal of glaucoma treatment is not to lower IOP but to maintain visual function. In this respect, VF evaluation is important, and while no significant change in MD was observed before and after the surgery, PSD was significantly smaller after the surgery. However, the VFs have not been sufficiently examined to draw a reliable regression line and a longer-term follow-up is needed.

In conclusion, we demonstrated the efficacy and safety of type P-50 Ex-PRESS surgery for open-angle glaucoma for a 3-year follow-up period. It was suggested that bleb needling in the late postoperative period would reduce the likelihood of achieving IOP under 12 mmHg. Longer-term follow-up is mandatory, and IOP control management, such as laser suture lysis, digital ocular compression and even bleb needling in the early postoperative period, should be performed to keep the IOP as low as possible to avoid making the adhesion between the sclera and the conjunctiva to keep the functional bleb.

## References

[1] Sawchyn AK, Slabaugh MA (2016). Innovations and adaptations in trabeculectomy. Curr Opin Ophthalmol.

[2] Beckers HJ, Kinders KC, Webers CA (2003). Five-year results of trabeculectomy with mitomycin C. Graefes Arch Clin Exp Ophthalmol.

[3] Shaarawy T, Goldberg I, Fechtner R (2015). EX-PRESS glaucoma filtration device: review of clinical experience and comparison with trabeculectomy. Surv Ophthalmol.

[4] Dahan E, Carmichael TR (2005). Implantation of a miniature glaucoma device under a scleral flap. J Glaucoma.

[5] Beltran-Agullo L, Trope GE, Jin Y, Wagschal LD, Jinapriya D, Buys YM (2015). Comparison of visual recovery following ex-PRESS versus trabeculectomy: results of a prospective randomized controlled trial. J Glaucoma.

[6] Wang W, Zhang X (2014). Meta-analysis of randomized controlled trials comparing EX-PRESS implantation with trabeculectomy for open-angle glaucoma. PLoS One.

[7] Sugiyama T, Shibata M, Kojima S, Ueki M, Ikeda T (2011). The first report on intermediate-term outcome of Ex-PRESS glaucoma filtration device implanted under scleral flap in Japanese patients. Clin Ophthalmol (Auckland, NZ).

[8] Kato N, Takahashi G, Kumegawa K, Kabata Y, Tsuneoka H (2015). Indications and postoperative treatment for Ex-PRESS(®) insertion in Japanese patients with glaucoma: comparison with standard trabeculectomy. Clin Ophthalmol (Auckland, NZ).

[9] Ishida K, Moroto N, Murata K, Yamamoto T (2017). Effect of glaucoma implant surgery on intraocular pressure reduction, flare count, anterior chamber depth, and corneal endothelium in primary open-angle glaucoma. Jpn J Ophthalmol.

[10] Ishida K, Moroto N, Murata K, Yamamoto T (2018). Publisher correction to: effect of glaucoma implant surgery on intraocular pressure reduction, flare count, anterior chamber depth, and corneal endothelium in primary open-angle glaucoma. Jpn J Ophthalmol.

[11] Aihara M, Kuwayama Y, Miyata K, Ohtani S, Ideta R, Hashimoto Y, Sasaki N, Shirato S (2019). Twelve-month efficacy and safety of glaucoma filtration device for surgery in patients with normal-tension glaucoma. Jpn J Ophthalmol.

[12] Netland PA, Sarkisian SR, Moster MR, Ahmed II, Condon G, Salim S, Sherwood MB, Siegfried CJ (2014). Randomized, prospective, comparative trial of EX-PRESS glaucoma filtration device versus trabeculectomy (XVT study). Am J Ophthalmol.

[13] Good TJ, Kahook MY (2011). Assessment of bleb morphologic features and postoperative outcomes after Ex-PRESS drainage device implantation versus trabeculectomy. Am J Ophthalmol.

[14] Mendoza-Mendieta ME, López-Venegas AP, Valdés-Casas G (2016). Comparison between the EX-PRESS P-50 implant and trabeculectomy in patients with open-angle glaucoma. Clin Ophthalmol (Auckland, NZ).

[15] Wagschal LD, Trope GE, Jinapriya D, Jin YP, Buys YM (2015). Prospective randomized study comparing Ex-PRESS to trabeculectomy: 1-year results. J Glaucoma.

[16] Mietz H, Arnold G, Kirchhof B, Diestelhorst M, Krieglstein GK (1996). Histopathology of episcleral fibrosis after trabeculectomy with and without mitomycin C. Graefes Arch Clin Exp Ophthalmol.

[17] Holló G, Naghizadeh F (2014). High magnification in vivo evaluation of the mechanism of failure of an Ex-PRESS shunt implanted under the sclera flap. Eur J Ophthalmol.

[18] Tojo N, Hayashi A, Otsuka M (2018). Factors influencing the filtration-bleb volume after Ex-PRESS(®) surgery. Clin Ophthalmol (Auckland, NZ).

[19] Sihota R, Angmo D, Ramaswamy D, Dada T (2018). Simplifying “target” intraocular pressure for different stages of primary open-angle glaucoma and primary angle-closure glaucoma. Indian J Ophthalmol.

[20] Meyer AM, Rosenberg NC, Rodgers CD, Webel AD, Nguyen PT, Wilson MK, Harbie K, Blake CR, Bolch CA, Sherwood MB (2019). Attaining intraocular pressure of ≤10 mm Hg: comparison of tube and trabeculectomy surgery in pseudophakic primary glaucoma eyes. Asia Pac J Ophthalmol (Phila).

[21] Lim R (2022). The surgical management of glaucoma: a review. Clin Exp Ophthalmol.

[22] Bertrand V, Fieuws S, Stalmans I, Zeyen T (2014). Rates of visual field loss before and after trabeculectomy. Acta Ophthalmol.

[23] Nakajima K, Sakata R, Ueda K, Fujita A, Fujishiro T, Honjo M, Shirato S, Aihara M (2021). Central visual field change after fornix-based trabeculectomy in Japanese normal-tension glaucoma patients managed under 15 mmHg. Graefes Arch Clin Exp Ophthalmol.

[24] Shigeeda T, Tomidokoro A, Araie M, Koseki N, Yamamoto S (2002). Long-term follow-up of visual field progression after trabeculectomy in progressive normal-tension glaucoma. Ophthalmology.

[25] Mataki N, Murata H, Sawada A, Yamamoto T, Shigeeda T, Araie M (2014). Visual field progressive rate in normal tension glaucoma before and after trabeculectomy: a subfield-based analysis. Asia Pac J Ophthalmol (Phila).

[26] Maris PJ, Ishida K, Netland PA (2007). Comparison of trabeculectomy with Ex-PRESS miniature glaucoma device implanted under scleral flap. J Glaucoma.

[27] de Jong LA (2009). The Ex-PRESS glaucoma shunt versus trabeculectomy in open-angle glaucoma: a prospective randomized study. Adv Ther.

[28] Marzette L, Herndon LW (2011). A comparison of the Ex-PRESS™ mini glaucoma shunt with standard trabeculectomy in the surgical treatment of glaucoma. Ophthalmic Surg Lasers Imaging.

[29] Moisseiev E, Zunz E, Tzur R, Kurtz S, Shemesh G (2015). Standard trabeculectomy and Ex-PRESS miniature glaucoma shunt: a comparative study and literature review. J Glaucoma.

[30] Allan EJ, Jones JM, Ding K, Khaimi MA (2016). Outcomes of bleb revision with mitomycin C after Ex-PRESS shunt surgery. J Glaucoma.

[31] Freidl KB, Moster MR (2012). ExPRESS shunt surgery: preferred glaucoma surgery in residency training?. Surv Ophthalmol.

[32] Mariotti C, Dahan E, Nicolai M, Levitz L, Bouee S (2014). Long-term outcomes and risk factors for failure with the EX-press glaucoma drainage device. Eye (Lond).

[33] Inoue T, Kawaji T, Inatani M, Kameda T, Yoshimura N, Tanihara H (2012). Simultaneous increases in multiple proinflammatory cytokines in the aqueous humor in pseudophakic glaucomatous eyes. J Cataract Refract Surg.

